# Clozapine-induced agranulocytosis

**DOI:** 10.1007/s00277-020-04215-y

**Published:** 2020-08-20

**Authors:** Aleksandar Mijovic, James H. MacCabe

**Affiliations:** 1 Department of Haematological Medicine, King’s College Hospital, London, SE5 9RS UK; 2 Institute of Psychiatry, King’s College London, London, UK

**Keywords:** Clozapine, Agranulocytosis, Re-challenge, Ethnic neutropenia

## Abstract

Wider use of clozapine, one of the most effective antipshychotic drugs, is precluded by its propensity to cause agranulocytosis. Currently, clozapine is used for treatment-resistant schizophrenia, with mandatory blood count monitoring for the duration of treatment. Agranulocytosis occurs in up to 0.8% of patients and presents a significant medical challenge, despite decreasing mortality rates. In this paper, we review the epidemiology of clozapine-induced agranulocytosis (CLIA), advances in identifying genetic risk factors, and the preventive measures to reduce the risk of CLIA. We discuss the pathogenesis of CLIA, which, despite receiving considerable scientific attention, has not been fully elucidated. Finally, we address the clinical management and suggest the approach to clozapine re-challenge in patients with a previous episode of neutropenia. With a significant proportion of clozapine recipients in Western hemisphere being Black, we comment on the importance of recognizing benign ethnic neutropenia as a potential impediment to clozapine administration. This review aims to aid haematologists and psychiatrists to jointly manage neutropenia and agranulocytosis caused by clozapine.

## Introduction

Drug-induced agranulocytosis is a potentially life-threatening, idiosyncratic reaction characterized by a profound decrease in neutrophil count and susceptibility to infection. Among the many causative agents, antipsychotic drug clozapine occupies a unique place due to its role in treatment-refractory schizophrenia (TRS), where it is often the only effective treatment [1]. Clozapine-induced agranulocytosis (CLIA) is an obstacle to clozapine use in a much larger number of patients with schizophrenia.

Clozapine (CZP), a dibenzodiazepine atypical antipsychotic drug, was introduced for treatment of schizophrenia in Europe in 1971, rapidly gaining popularity due to its efficacy and virtual absence of extrapyramidal side effects [2]. However, its propensity to cause neutropenia and agranulocytosis was soon recognized [3], leading to its withdrawal. A double-blind, randomized trial, which demonstrated superior efficacy of CZP over chlorpromazine [4] in TRS, led to its reintroduction in 1989 in Europe and in 1990 in the USA. However, CZP use is largely restricted to treatment-resistant cases and blood count monitoring, mandatory for the entire duration of treatment, has been introduced in most countries.

The National Institute for Clinical Excellence 2014 guidelines state that CZP is the drug of choice for treatment-resistant psychosis, defined as failure to respond to at least two other trials of antipsychotic drugs (www.nice.org.uk/guidance/cg178, 2014). Treatment resistance, thus defined, affects approximately one-third of patients with schizophrenia. Annual cost of TRS has been estimated at $34 billion in the USA, 3–11-fold greater than that for treatment-responsive schizophrenia [5]. There is evidence that CZP is superior to any other antipsychotic in reducing hospitalizations [6] and all-cause mortality [7].

Despite the reduction of the incidence of CLIA due to blood monitoring programs, and reduced mortality from drug-induced agranulocytosis, psychiatrists may be reluctant to prescribe a drug with a potentially fatal side effect. Additional burden of regular blood testing discourages both patients and clinicians from choosing clozapine. Due to these concerns, CZP remains underutilized in patients with TRS [8].

In this article we review the epidemiology, pathogenesis, and management of CLIA and suggest the approach to CZP re-challenge in patients who have previously had neutropenia during CZP treatment.

## Epidemiology

Agranulocytosis, defined as neutrophil count of less than 0.5 × 10^9^/L (500/μL), developed in 0.8% (95% CI, 0.61 to 0.99) of clozapine-treated patients in the classic study by Alvir et al. [9] in the USA. This figure represented cumulative incidence of CLIA after 1 year, whereas the corresponding figure after one and a half years was 0.91%. Lambertenghi-Deliliers [10] found a similar incidence in Italy (0.7%). Interestingly, Tang et al. [11] reported a lower incidence (0.21%) in China where, unlike in Western countries, CZP is widely prescribed as a first-line antipsychotic.

A US review credited the establishment of the Clozaril (trade mark of Clozapine) National Registry, held by the drug manufacturer, for a much lower rate of agranulocytosis recorded over 5 years in over 99,000 treated patients [12]. The incidence of agranulocytosis in this study was 0.38%. A similar incidence of CLIA—0.4%—was reported by Li et al. [13] in a large meta-analysis of 36 studies comprising over 260,000 patients from various countries. Of note, incidence of this magnitude, i.e. approximately 1 in 250 patients, greatly exceeds the incidence of drug-induced agranulocytosis in general population, estimated to be 1.6–7.0 in 1 million [14, 15].

CLIA typically occurs within the first 18 weeks of treatment, with few cases occurring beyond 6 months [9]. After 1 year of treatment, residual risk is estimated at 0.39/1000 patient/years [16]. Occasional cases have been reported after several years of treatment [17]; in such cases, one needs to carefully consider other etiological causes of neutropenia.

Mortality due to CLIA decreased since the initial reports [2], with estimates ranging from 2.7 to 3.1% [9, 12]. This is lower than the mortality from all drug-induced agranulocytosis, estimated at about 7-10% [15, 18]. This difference, if proved genuine, may be due to younger age and fewer comorbidities of CZP-treated patients, in comparison with the patient population exposed to other drugs associated with agranulocytosis, such as antibiotics, anti-inflammatory drugs, and anti-thyroid drugs. It can be argued that regular blood monitoring leads to early detection of agranulocytosis and CZP discontinuation, before infection is established, thus reducing fatalities.

## Risk factors and genetics

Alvir et al. [9] found that the risk of CLIA was slightly higher in females (RR 1.60, 95% CI 0.99–2.58, after adjustment for age). Neither Lambertenghi-Deliliers [10] nor Stubner et al. [19] could confirm these findings. Age also increases the risk of CLIA [15, 20]. There is also evidence from the large study of Munro et al. [20] that the risk in Asian individuals is 2.4-fold higher than in Caucasians.

Genetic basis of CLIA was postulated after early investigations by Yunis et al. [21], who found a strong association between the *HLA-B38*, DRB1*0402, DRB4*0101, and DQB1*0302 haplotype with CLIA in Ashkenazi Jews. It was subsequently suggested that this association is due to a linkage disequilibrium between HLA class II genes and polymorphisms of other genes within the MHC complex, namely the heat shock protein *HSP70-2* [22], and the tumour necrosis factor gene [23], but this hypothesis still awaits confirmation.

More recently, Athanasiou et al. [24] sequenced 74 candidate genes from a cohort of CLIA patients and controls, and then tested the significant markers in a second patient/control cohort. They found that the single nucleotide polymorphism 6672G>C of the *DQB1* gene conferred 16.9-fold greater odds of having CLIA, compared with patients without this variant. Their findings were replicated in an independent sample using genome-wide association study [25], and currently represent the most robust genomic association with CLIA.

Additionally, Legge et al. [25] identified the association of an intronic polymorphism of two hepatic transporter genes (*SLCO1B3* and *SLCO1B7*) with CLIA, with an odds ratio of 4.32 (*p* = 1.79 × 10^−8^). Moreover, SNPs of these genes had already been implicated in adverse drug reactions, including docetaxel-induced neutropenia.

Genetic aetiology of neutropenia/agranulocytosis induced by CZP is complex and is likely to involve variants of several genes including *HLA-DQB1*, *HLA-B*, and *SLCO1B3/SLCO1B7* [26]. Further studies are required to separate causal variants from association signals and to improve our understanding of mechanisms underlying these associations.

## Pathogenetic mechanisms

Although CLIA received considerable scientific attention over the last 25 years, its pathogenesis remains unclear. CLIA is an idiosyncratic phenomenon, affecting only a small subset (about 1%) of exposed subjects. Therefore, it is not surprising that CLIA is not dose-related [9]. Studies that addressed immune-mediated mechanisms using semi-solid culture systems [27] or flow cytometry [28] failed to demonstrate the presence of antibodies against neutrophils or their precursors. Guest et al. [29] investigated the toxicity of CZP and its metabolites on neutrophils and their precursors (CFU-GM) in patients with CLIA, clozapine-associated neutropenia, and asymptomatic patients on CZP: the results were inconsistent; importantly, they were unable to detect T-lymphocyte clones specific for CZP metabolites bound to leukocyte antigens. Recently, Regen et al. [30] applied an adapted lymphocyte proliferation assay in patients with a history of CLIA, those on CZP without haematological disorders, and healthy controls. They were able to show significantly raised lymphocyte proliferation rates only in patients with the history of CLIA but not in the other two groups. While this study lends credence to the immune pathogenesis of CLIA, triggered by the presence of the drug or its metabolite, its predictive value and clinical applicability are unproven at present.

Gerson et al. [31] tested the main CZP metabolites, N-desmethylclozapine and N-oxide clozapine, in the semisolid progenitor culture system and found that N-desmethylclozapine was toxic to granulocyte progenitors (CFU-GM) (and also to erythroid (BFU-E) and multipotent progenitor cells (CFU-GEMM)), at concentrations 3–6-fold higher than those normally achieved during treatment. They hypothesized that the toxicity of N-desmethylclozapine may be compounded by another metabolic intermediate, such as *nitrenium ion*. Uetrecht et al. [32] found evidence that CZP is metabolized, by the action of myeloperoxidase, to a nitrenium ion, a short-lived but highly reactive metabolite. The pivotal role of myeloperoxidase in this metabolic step could explain why CZP and its metabolites affect almost exclusively granulocytes and their precursors. Nitrenium ion binding triggers apoptosis of granulocytic cells [33, 34], possibly modified by genomic factors that determine individual susceptibility to apoptosis, such as variants of the heat shock protein and/or tumour necrosis factor genes [22, 23]. Another putative mechanism of CLIA, not mutually exclusive with apoptosis induction, is that the irreversible binding of nitrenium ion, with consequent alterations of granulocyte membrane, leads to a formation of neo-antigen(s) that elicit an immune response [34, 35].

In keeping with its pivotal role in the pathogenesis of CLIA, nitrenium ion formation can also be catalysed by flavin-containing monooxygenase-3 (FMO3), an enzyme abundant in human liver, with a major role in metabolism of many foods and drugs, including CZP [36].

## Prevention

To reduce the occurrence of agranulocytosis, centralized mandatory haematological monitoring for patients on CZP was introduced in many countries. In the UK, after registration with one of the three monitoring services, the patient must have full blood counts weekly in the first 18 weeks of treatment, then fortnightly until week 52, and then monthly for the duration of treatment (bnf.nice.org.uk/drug/clozapine, 2020). All UK monitoring services classify the white blood cell (WBC) and absolute neutrophil counts (ANC) into three zones (Table 1), guiding patient’s further management. In a similar fashion, in the USA, full blood counts are monitored weekly for the first 6 months, fortnightly in the second 6 months of treatment, and monthly thereafter. In 2015, the Food and Drug Administration (FDA) announced centralization of the monitoring systems into a “risk evaluation and mitigation strategy” (REMS) and, in parallel, promoted the use of ANC as the only parameter for monitoring patients on CZP. Thresholds for CZP discontinuation in the USA are lower than those currently used in the UK by 0.5 × 10^9^/L (Table 1); thus, CZP treatment is interrupted if ANC is less than 1000/μL. In patients with benign ethnic neutropenia (BEN), CZP is discontinued when neutrophils fall below 500/μL (= 0.5 × 10^9^/L) (www.fda.gov/drugs/drugsafety, 2019).

**Table 1:** UK and US clozapine monitoring criteria

Zone	WBC	ANC	ANC under BEN criteria	Guidance
Green	➢ 3.5	➢ 2.0	> 1.5	CZP safe to be dispensed or continue
Amber	3.0-3.5	1.5-2.0	1.0-1.5	Continue, but monitor twice weekly
Red	<3.0	<1.5	N<1.0	STOP
FDA 2015	-	<1.0	<0.5	STOP

In order to assess the potential impact of the relaxation of FDA criteria on CZP treatment, Sultan et al. [37] retrospectively evaluated data collected between 1999 and 2012. Among 246 patients treated with clozapine, five patients required interruption of treatment; if new FDA criteria had been applied, just one patient would have discontinued treatment. Importantly, no cases of agranulocytosis were observed in the entire study group.

## Clinical picture and management

Typically, agranulocytosis presents with fever, mouth ulcers, and sore throat, although some patients remain entirely asymptomatic despite very low neutrophil counts. Agranulocytosis is, somewhat arbitrarily, defined as ANC < 0.5 × 10^9^/l; typically, haemoglobin and platelet count are normal but may decrease later due to septicaemia. Bone marrow aspiration/biopsy is not routinely performed in psychiatric patients, but in five cases seen by one of us (AM), the marrow aspirate was virtually devoid of granulocytic precursors, with only an occasional myeloblast or promyelocyte on the film.

CLIA usually develops several weeks after the commencement of treatment. Andersohn et al. [38] reported a median interval of 56 days, and the average duration of agranulocytosis of 12 days. Following re-challenge, time from re-treatment to the onset of CLIA may be shorter than in the first episode [39]. Use of granulocyte colony-stimulating factor (GCSF) may reduce the duration of agranulocytosis by 4 to 5 days; in a systematic review of the use of GCSF in CLIA, Lally et al. [40] reported the mean time from starting GCSF to recovery of 7 days.

If agranulocytosis develops, CZP must be stopped immediately. In reality, CZP will usually have already been stopped, in conformance with the monitoring criteria. Careful clinical and microbiological monitoring should be instituted. We prefer to transfer the patient with CLIA to a haematology unit with isolation facility and clean-air system, but if the patient remains on a psychiatric ward, it is of paramount importance to have haematology input available 24 h a day. Despite the paucity of high-quality evidence that GCSF shortens the duration of neutropenic phase, we recommend starting GCSF, e.g. Filgrastim (or a biosimilar) 300 μg or Lenograstim 263 μg SC daily. If the patient becomes febrile, or develops signs of sepsis or a focal infection, antibiotics should be instituted promptly, following local or national guidelines for the management of febrile neutropenia.

Individual patients, who do not have signs of infection and have good support at home, may be counselled about protective measures, given GCSF, and followed in outpatient clinic until neutrophil recovery.

About 1–3% of patients on CZP develop mild/moderate neutropenia, which may or may not progress to agranulocytosis [36, 41]. Clinicians treating patients with CZP should bear in mind that “simple” neutropenia on CZP may be brought about by the concomitant use of other neutropenia-inducing drugs, including antibiotics [36, 42] and sodium valproate [43, 44], or by a concomitant viral infection.

## Re-challenge with clozapine

Once a patient develops a “red” neutrophil count (< 1.5 × 10^9^/l or < 1.0 × 10^9^/l in BEN patients in the UK), they will be deemed non-re-challengeable and registered in a central database shared across all monitoring systems. Nevertheless, in view of the unique efficacy of CZP, psychiatrists may undertake a re-challenge on an “off-label” basis. Several studies in the UK have addressed this matter. Dunk et al. [39] reported that 20/53 (38%) patients they studied after re-challenge experienced a second blood dyscrasia, of whom 9 developed agranulocytosis. This also meant that 62% of their patients would have been unnecessarily denied CZP, had re-challenge not been attempted. Kanaan and Kerwin [45] reported 25 re-challenges with co-prescribed lithium and found that only 1 (4%) developed a second dyscrasia, indicating that lithium can be a useful adjunct in CZP re-challenge. A more recent study from our group [43] found that 79% of CZP re-challenges were successful. Of note, none of the 19 patients in our study, similar to the study of Dunk et al. [39], had agranulocytosis in the first episode. In keeping with these observations, a systematic review found that 80% of re-challenges *that followed agranulocytosis in the first episode* were unsuccessful [46].

In summary, re-challenge with CZP is an option for patients with resistant schizophrenia, especially if CZP was discontinued because of leukopenia or moderate neutropenia, but not agranulocytosis. In any case, re-challenge should be conducted with a management plan agreed in close collaboration with a haematologist. We believe that lithium and GCSF have a role in supporting re-challenge. Lithium may be useful, especially if the patient has a mood disorder for which lithium is also indicated, although it should be noted that lithium itself has non-negligible toxicities. Medication should be reviewed and drugs with potential to cause neutropenia (e.g. sodium valproate, carbamazepine) should be discontinued, and their substitution with drugs with little or no neutrophil toxicity (e.g., levetiracetam), should be considered.

If re-challenge fails, continuing CZP is hazardous. Data are somewhat conflicting about the incidence of neutropenia due to other antipsychotic drugs: Stubner et al. [19] found a significantly higher incidence of neutropenia with CZP and also perazine, compared with haloperidol, risperidone, and promethazine; conversely, Myles et al. [47] asserted that CZP does not have a stronger association with neutropenia than other antipsychotic drugs. Our clinical experience, supported by literature search, suggests that severe neutropenia is exceedingly rare with Haloperidol and Amisulpride, and virtually non-existent with Aripiprazole.

## Clozapine and benign ethnic neutropenia

BEN is defined as neutropenia (< 1.5 × 10^9^/L) with no apparent cause in individuals of African or Afro-Caribbean descent, but also some Arab ethnic groups and Yemenite Jews [48, 49]. As the name suggests, BEN does not confer an increased risk of infection. Recent molecular studies have associated low neutrophil counts in people of African ancestry with the − 46 T > C substitution in the non-coding region of the Duffy antigen receptor for chemokines (*DARC,* also known as *ACKR1*) gene. Homozygosity for this mutation (C/C genotype) results in the Duffy (Fy)—negative (a− b−) red cell phenotype. The *DARC/ACKR1* gene encodes a receptor that binds a number of chemokines; some of which enhance neutrophil recruitment to sites of inflammation and movement across the vascular endothelium [50, 51].

Estimates of the prevalence of BEN in black people differ. Hsieh et al. [49] found neutrophil counts of < 1.5 × 10^9^/L in only 4.5% of Black Americans. Elsewhere, BEN prevalence appears appreciably higher—25–40% [48, 52]. This discrepancy could be due in part to different thresholds used for defining neutropenia (i.e. 1.5 vs 2.0 × 10^9^/L), but also to factors such as racial admixture, or different methods of ascertaining ancestry.

BEN has been a diagnosis of exclusion, based on patient’s ethnic background and lack of other potential causes of neutropenia. Serological finding of Fy (a− b−) red cell phenotype corroborates the diagnosis of BEN, but it is more useful for its negative predictive value (AM, unpublished data). It is plausible that *ACKR1* genotyping become routine, but, until it does, simple clinical and laboratory tests can provide a satisfactory alternative.

The clinical impact of BEN in the context of CZP treatment is exemplified by the fact that about a half of CZP recipients in the large area served by the South London and Maudsley NHS Trust are black [53]. There is evidence that BEN is underdiagnosed and that, despite the introduction of modified ZTAS criteria for BEN (Table 1), black patients are more likely to discontinue clozapine than their white counterparts [53, 54], leading to CZP underutilization in this patient population.

## Conclusions

Agranulocytosis is one of the most serious complications of clozapine treatment, and the burden of haematological monitoring leads to under-utilization of clozapine, with significant medical and economic consequences. In public health terms, the significance of CLIA greatly exceeds that of any other drug-induced agranulocytosis.

Key messages of this paper are summarized in the box below:

**Table:** 

1. Incidence of CLIA has diminished due to haematological monitoring of patients on CZP. 2. Despite current trends toward partial relaxation of monitoring criteria, CZP remains under-utilized, especially in patients of Black racial background. 3. Haematological input can minimize complications, avoiding delays in referrals and unnecessary clinic visits through establishment of “virtual clinics.” 4. Management of CLIA requires close surveillance and prompt treatment, preferably in institutions with suitable facilities and experience in the treatment of neutropenic sepsis. 5. Re-challenge with CZP is often successful but ought to be carried out with robust management plans and close inter-disciplinary collaboration. 6. Safety of CZP may be enhanced by further elucidation of genetic risk factors.	
